# Reply to locked vs. unlocked AED cabinets: The Western Australian perspective on improving accessibility and outcomes

**DOI:** 10.1016/j.resplu.2024.100838

**Published:** 2024-12-14

**Authors:** Janet E. Bray, Gavin D. Perkins

**Affiliations:** Monash University, Melbourne, Australia; University of Warwick, Warwick, United Kingdom

Dear Editor,

We thank Mr Didcoe and Colleagues for sharing their thoughts on the issue of locked automated external defibrillator (AED) cabinets.^1^ We appreciate their interest in our scoping review,^2^ and understand their concerns regarding the challenges posed by unlocking AED cabinets and the need to balance security against rapid access.

While we found no data to demonstrate open access to AEDs is associated with increased use or improved outcomes, our review does show a locked AED cabinet does not guarantee an AED will not be stolen or vandalised.^3^ As seen in a recent photo captured by an author (Fig. 1), breaking into a locked AED by smashing the cabinet and the potential for injury is not limited to one type of cabinet and appears to also occur in cabinets locked by a keypad.

**Fig. 1:**
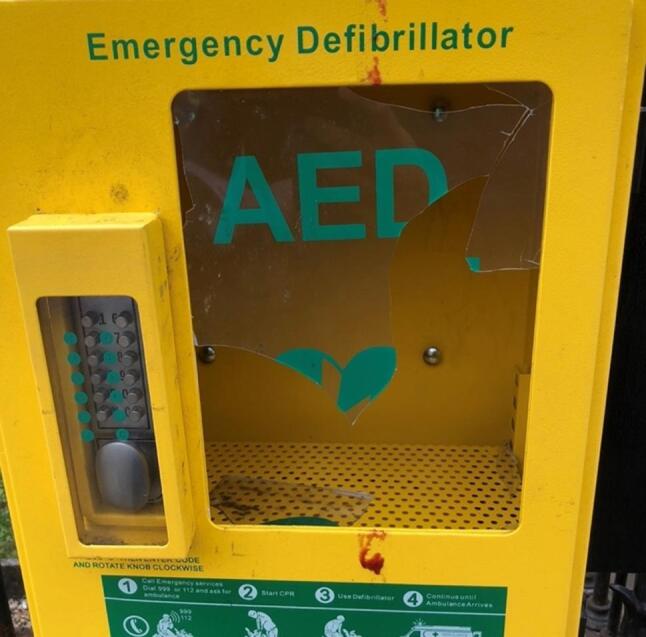
Photo of an automated external defibrillator (AED) cabinet captured by the authors.

The other issue with AED cabinets locked with keypads is the logistics of accessing the code. While calling emergency medical services (EMS) is a critical component in the out-of-hospital cardiac arrest chain of survival, unlocking an AED via a code held by dispatch requires access to a phone at the AED ‘s location and may result in crucial lost minutes while accessing a dispatcher and retrieving the code. Research from your own region does not describe the need for a code or phone when a dispatcher asks the caller if an AED is available and to retrieve.^4^ Does access, therefore, require an additional call to EMS and a further explanation of why it is needed? Recent evidence indicates rapid defibrillation is crucial for successful resuscitation, with every minute of delay in delivering the first defibrillation reducing the chance of terminating ventricular fibrillation (VF), an increased likelihood of VF reoccurrence, and poorer patient outcomes.^5^

The recent treatment recommendations by the International Liaison Committee on Resuscitation advise against using locked AED cabinets but also advise that access controls must ensure minimal delays if locked cabinets are used.^6^ We welcome further research in this area to guide future use and recommendations.

We applaud Mr Didcoe and Colleagues’ commitment to ensuring that AEDs are available and functional when needed. Thank them again for contributing to this important conversation, and we welcome continued dialogue to find solutions that optimise accessibility and security.

## CRediT authorship contribution statement

**Janet E. Bray:** Writing – review & editing, Writing – original draft. **Gavin D. Perkins:** Writing – review & editing.

## Funding

JEB is funded by a Heart Foundation Future Leader Followship.

## Declaration of competing interest

The authors declare the following financial interests/personal relationships which may be considered as potential competing interests: The authors are Editors of Resuscitation Plus. JB has grant funding from the Laerdal Foundation.
